# Microbial Profiles of Retail Pacific Oysters (*Crassostrea gigas*) From Guangdong Province, China

**DOI:** 10.3389/fmicb.2021.689520

**Published:** 2021-07-07

**Authors:** Mingjia Yu, Xiaobo Wang, Aixian Yan

**Affiliations:** Department of Food Science, Foshan Polytechnic, Foshan, China

**Keywords:** Pacific oysters, 16S ribosomal DNA amplification sequencing, microbial diversity, *Vibrio vulnificus*, *Crassostrea gigas*

## Abstract

Oysters are one of the main aquatic products sold in coastal areas worldwide and are popular among consumers because of their delicious taste and nutritional value. However, the microorganisms present in oysters may pose health risks to consumers. In this study, the microbial communities of Pacific oysters (*Crassostrea gigas*) collected from aquatic product markets in three cities (Guangzhou, Zhuhai, and Jiangmen) of Guangdong Province, China, where raw oysters are popular, were investigated. The plate counts of viable bacteria in oysters collected in the three cities were all approximately 2 log colony-forming units/g. High-throughput sequencing analysis of the V3–V4 region of the 16Sribosomal DNA gene showed a high level of microbial diversity in oysters, as evidenced by both alpha and beta diversity analysis. Proteobacteria, Bacteroidetes, and Firmicutes were the dominant phyla of the microorganisms present in these samples. A variety of pathogenic bacteria, including the fatal foodborne pathogen *Vibrio vulnificus*, were found, and *Vibrio* was the dominant genus. Additionally, the relationship between other microbial species and pathogenic microorganisms may be mostly symbiotic in oysters. These data provide insights into the microbial communities of retail oysters in the Guangdong region and indicate a considerable risk related to the consumption of raw oysters.

## Introduction

Oysters are one of the most commonly farmed shellfish in China, with a total output of approximately 5.14 million tons in 2018 (Fisheries administration of Ministry of Agriculture and Rural affairs of the People’s Republic of China, 2019). They are considered a nutrient-rich source of proteins, unsaturated fatty acids, vitamins, and minerals (Ackman, 1990; Cabello et al., 2004; Caglak et al., 2008; Cruz-Romero et al., 2008a; Rey et al., 2012). The Pacific oyster is the main oyster species sold in the coastal areas of southern China. However, similar to most seafood with abundant water and nutrient contents, oysters tend to undergo spoilage during transportation and storage. Microbial proliferation results in spoilage and unacceptable quality during storage (Gram and Dalgaard, 2002; Boziaris and Parlapani, 2017).

In addition to the deterioration of the quality of oysters caused by spoilage, the risks to human health attributed to pathogenic microorganisms present in raw oysters have attracted considerable attention. Oysters are one of the few animal foods that are consumed whole and raw by humans. The consumption of raw oysters has become popular in Asia, including China. Thus, the safety of a product is vital to consumer health (Froelich and Noble, 2016). *Pseudomonas* and *Vibrio* were found to be the predominant bacteria in spoiled Pacific oysters bred by conventional cultivation (Cao et al., 2010; Cruz-Romero et al., 2008b). *V. vulnificus* and *V. parahaemolyticus* are the most common types of life-threatening foodborne pathogens and have been reported in *Vibrio-*associated wound infections (Froelich and Noble, 2016). *Vibrio* infections associated with eating uncooked or undercooked oysters continue to increase (Huss et al., 2014). In the US, *V. vulnificus* is the most fatal foodborne pathogen, and it is responsible for 95% of all seafood-related deaths. Even with aggressive medical treatment, its fatality rate are about 50%. These bacteria also have a second route of infection. They can enter the body via wounds, either preexisting or related to activities such as seafood handling or oyster harvesting (Jones and Oliver, 2009; Letchumanan et al., 2014; Froelich and Noble, 2016). Additionally, the leading causes of foodborne gastroenteritis in Japan include *V. parahaemolyticus* infection. This infection affects more than 10,000 individuals in 500–800 outbreaks annually (Food and Agriculture Organization, 2011). This pathogen is estimated to account for half of the foodborne illnesses reported in Asian countries (Chen et al., 2018). In the Guangdong province of China, the current trend suggests that raw oysters are consumed more frequently by an increasing proportion of the population, which results in new food safety problems. Individuals with impaired immune systems, children, pregnant women, and older adults are among the most susceptible to foodborne infections (Lund and O’Brien, 2011). Therefore, the microbial diversity of oysters at the consumer terminal is very important for food hygiene and safety.

To evaluate the quality of retail Pacific oysters in the Guangdong region and to assess their potential health risks to humans, we collected Pacific oysters sold in three coastal cities (Guangzhou, Zhuhai, and Jiangmen) in Guangdong Province. High-throughput sequencing was performed to determine the bacterial profiles of the samples. These data will offer insight into the microbial status of Pacific oysters in this area and may provide a reference for establishing targeted risk reduction strategies for oyster consumption.

## Materials and Methods

### Sample Collection

Oysters were collected from three cities, namely Zhuhai, Guangzhou, and Jiangmen, of Guangdong Province, China. The fresh oysters were purchased from two markets in Guangzhou, three markets in Jiangmen and one market in Zhuhai, and at least four different stalls in each market were selected (Supplementary Figure 1). The oysters in markets of Guangzhou were transported from other places, and those in markets of Jiangmen and Zhuhai were obtained locally (Supplementary Table 1). Every two oysters randomly purchased from the same stall were mixed into one sample to exclude sampling deviation, and the accumulated 52 samples from different stalls were divided into 3 groups depending on the cities from which they were collected. All oyster samples were rinsed with tap water to remove contaminants, such as dirt, on the surface.

### Microbiological Assays

Total viable count (TVC) assays were conducted as per previously described methods with slight modifications (Sanna et al., 2018). Ten grams of each sample was weighed and placed in a sterile homogenizing bag containing 90 mL of normal saline. In the homogenizer, the sample was subjected to flapping for 2.5 min to prepare a 1:10 homogenate (1 mL of homogenate was collected using a pipette and outsourced for DNA extraction and sequencing). A suitable diluent for plate count analysis was selected and cultured for 48–72 h. Clones was counted when the cells were visible.

### DNA Extraction

DNA was extracted from the homogenate of the oysters using a DNA extraction kit (Magen Hipure Soil DNA Kit, Angen Biotech, Guangzhou, China) according to the manufacturer’s instructions, and quality control was performed using the Qubit^®^ DSDNA HS Assay Kit (Thermo Fisher Scientific, MA, United States).

### 16Sribosomal DNA (rDNA) Amplification Sequencing

The Qubit 3.0 Fluorometer (Thermo Fisher Scientific, Waltham, MA, United States) was used to control the quality of the DNA, which was subsequently used to perform amplification of the V3–V4 hypervariable regions of prokaryotic16S rDNA. Pairing primers were designed by GENEWIZ (South Plainfield, NJ, United States). The sequence of the forward primer was “CCTACGGRRBGCASCAGKVRVGAAT,” and the reverse primer contained the sequence “GGACTACNVGGGTW TCTAATCC.” Each polymerase chain reaction volume was 25 μL, containing 2.5 μL of TransStart Buffer (TransGen, Beijing, China), 2 μL of dNTPs, 1 μL of each primer, and 20–30 ng of template DNA. Then, the indexed adapters were attached to the ends of the amplicons to generate indexed libraries for subsequent next-generation sequencing using the Illumina platform (San Diego, CA, United States). The libraries were validated using the Qubit3.0 Fluorometer (Thermo Fisher Scientific) and quantified to 10 nmol. The Illumina MiSeq instrument was used to load and sequence the DNA libraries according to the manufacturer’s instructions. Sequencing was performed under the paired-end 250-bp mode. The raw reads were trimmed using the Cutadapt software to generate clean reads.

### Microbial Diversity Analysis

Data with clean reads were analyzed by using the nf-core/ampliseqv1.2.0 pipeline, and the microbial diversity within our datasets were determined by setting the optional parameters “- -multiple Sequencing Runs,” “- -trunclenf 220,” and “- -trunclenr 180” (to resemble the truncation values of QIIME2 with DADA2) (Straub et al., 2020). The 16s rRNA gene comparison database, SILVA v132 (Quast et al., 2013), was used to perform clustering at 99% similarity. Based on the species annotation and abundance of effective operational taxonomic units (OTUs), functional annotations were obtained based on Kyoto Encyclopedia of Genes and Genomes pathway analysis using PICRUSt2 (Douglas et al., 2020).

### Phylogenetic Analysis

The OTU sequences of potential pathogenic genera and the reference 16S rRNA sequences of corresponding pathogenic species were aligned by using MUSCLE v3.8.1551 and the parameter of “-maxiters 2” (Edgar, 2004). Maximum likelihood phylogenetic trees were constructed with the best-fit substitution model implemented in IQ-TREE v2.0.3 (Nguyen et al., 2015).

### Microbiome Interaction Analysis

The interaction network of bacteria within the microbiome was estimated at the genus level according to the Sparcc method (You et al., 2019). Connected retention data were saved with satisfaction of the *P-*value < 0.05 and correlation value > 0.6 thresholds in both methods. The interaction networks were visualized using the Cytoscape 3.7.2 software (Shannon et al., 2003).

## Results

### Bacterial Richness and Diversity

**FIGURE 1:**
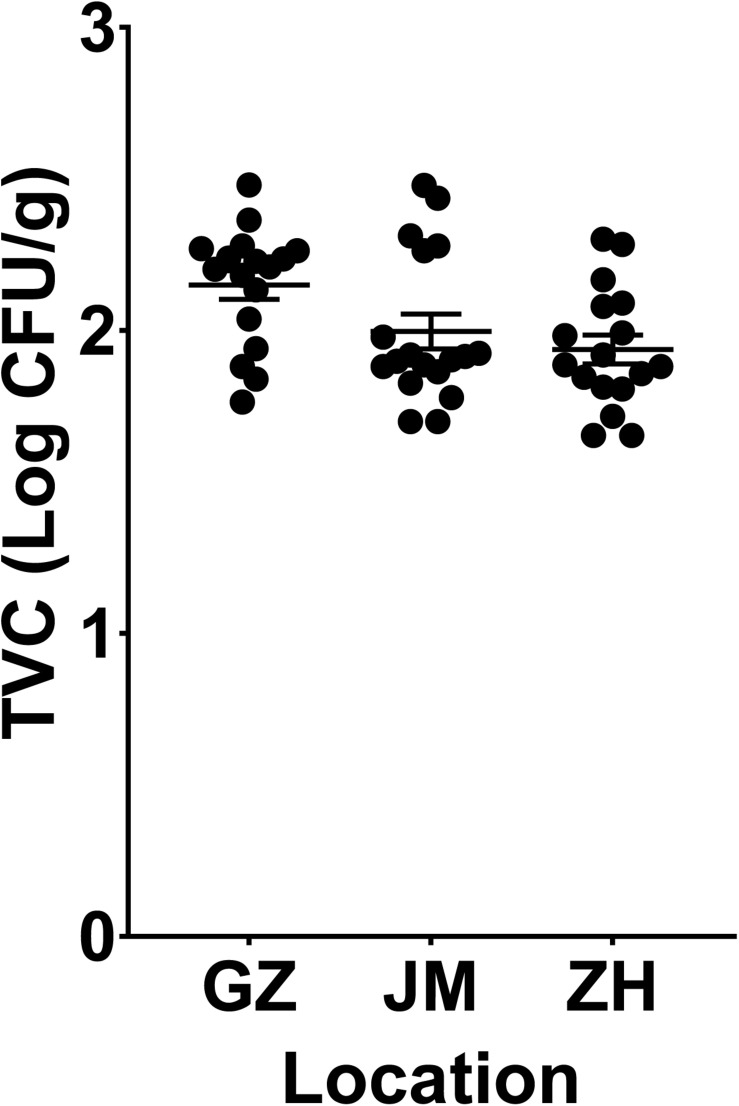
Evaluation of the total viable bacteria count in oyster samples collected from three cities in Guangdong Province, China. GZ, Guangzhou; JM, Jiangmen; ZH, Zhuhai.

According to the TVC assay results, all plate counts of viable bacteria in oysters were approximately 2 log colony-forming units (CFU)/g. The samples from Guangzhou (GZ) have the highest richness of viable bacteria, and it showed no significant difference compared to the samples from Jiangmen (JM) and but a significant difference compared to those from Zhuhai (ZH) (*P* < 0.05, Figure 1). To further determine the microbial diversity in oysters, we analyzed the diversity of the microbiome within these samples. An average of 65,000 clean sequence reads, ranging from 40,691–105,248, were obtained for each sample. These clean sequences were clustered into 6,025 OTUs whose similarity was >99% (Supplementary Table 2).

**FIGURE 2:**
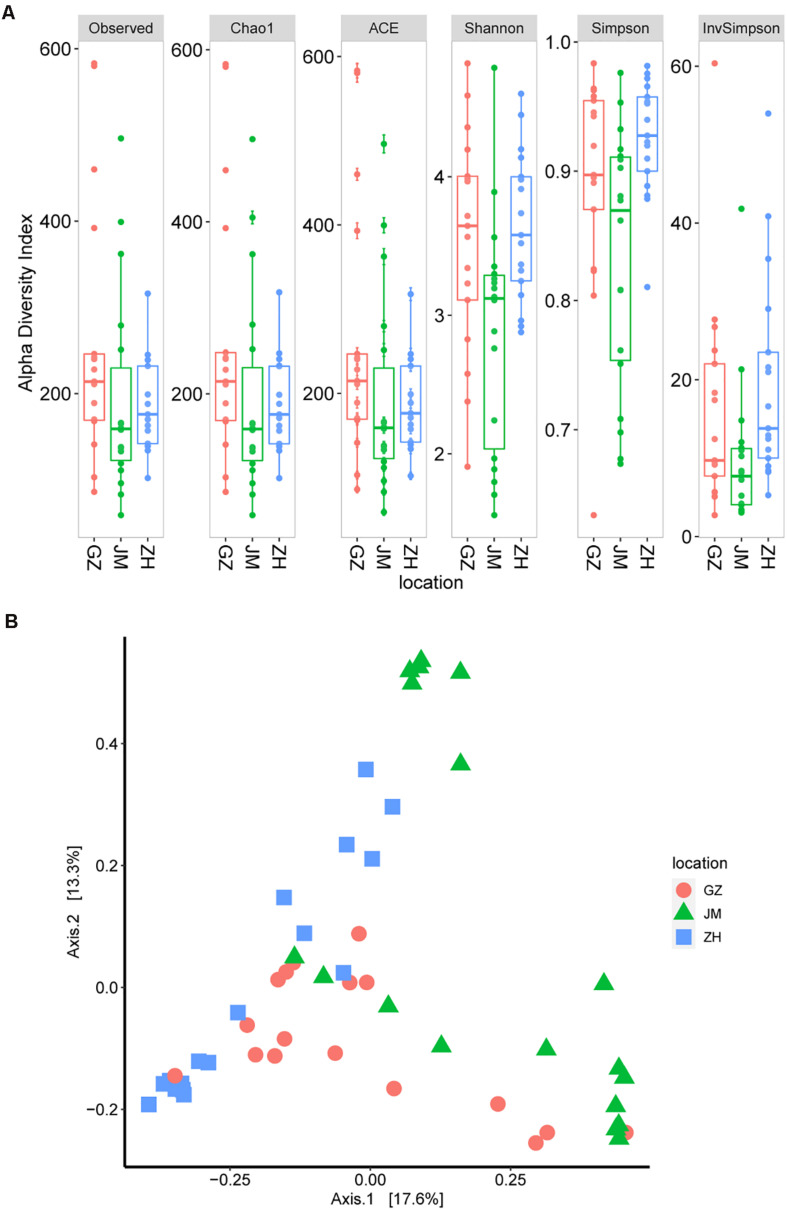
Microbiome diversity in oyster samples. **(A)** Boxplot of alpha diversity measured by the observed operational taxonomic units as well as theChao1, ACE, Shannon, Simpson, and InvSimpson indices. **(B)** Principal coordinate analysis plot illustrating the data based on beta-diversity metrics measured by using the Bray–Curtis distance.

We then estimated the alpha diversity of these samples according to the observed OTUs as well as the Chao1, ACE, Shannon, and Simpson indices. The results showed that the oyster samples from Guangzhou had the highest alpha diversity (Figure 2A). Additionally, the samples from Jiangmen had a significantly lower alpha diversity than those from Guangzhou (*P* = 0.029; Kruskal–Wallis test for the Shannon index), and these findings were different from the TVC assay results. This discrepancy could be easily inferred because the 16S rRNA sequencing revealed the total bacterial species information, whereas the TVC assay results only reflected the viable bacteria (Caporaso et al., 2010). Based on the greater accuracy of OTU estimates, the species abundance of oyster samples from different cities may show a difference, and the transported samples in Guangzhou seemed to be more complicated than those locally obtained samples.

The high diversity of microbial communities in oysters could also be inferred from the beta diversity index. According to the principal coordinate analysis of beta diversity, which is generally measured by using the Bray–Curtis distance (Pannaraj et al., 2017), no evident clusters were observed across geographical differences (Figure 2B). These results suggest the existence of a high level of bacterial diversity in oyster samples collected from different cities or even in those within the same city.

### Composition of the Bacterial Community

**FIGURE 3:**
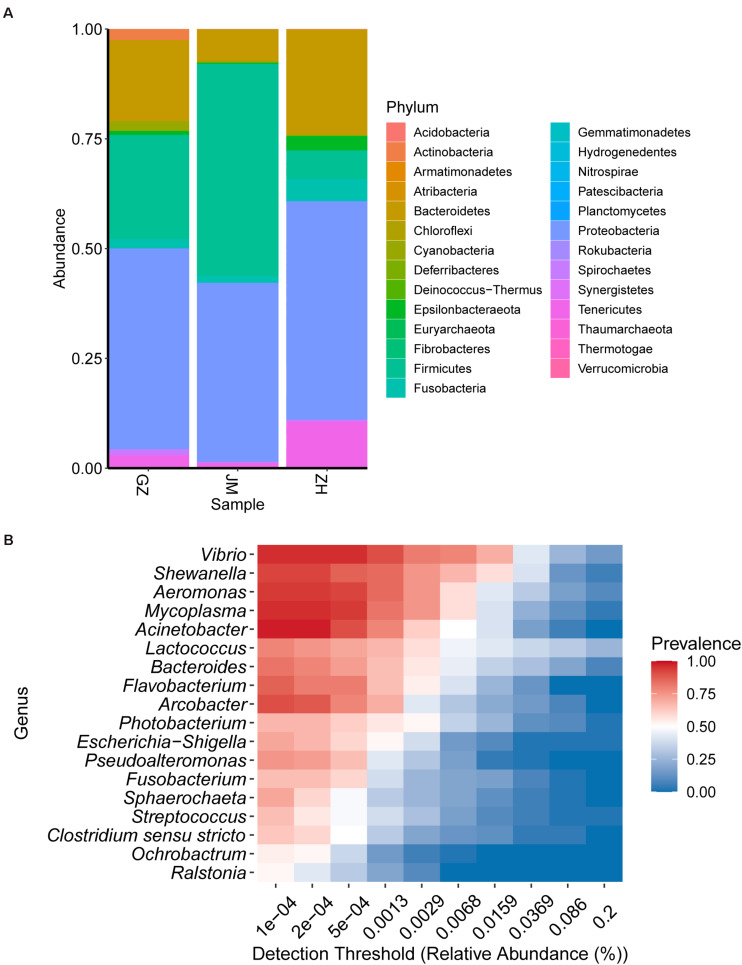
Bacterial community structure in oyster samples. **(A)** The relative abundance of bacterial species at the phylum level. **(B)** Heatmap illustrating the distribution of the major genera among all samples.

The composition of the bacterial community was studied at the phylum and genus levels of classification. At the phylum level, members of Proteobacteria predominant in oysters collected across different cities; however, Firmicutes, Bacteroidetes, and Tenericutes were the most variable phyla (Figure 3A). The abundance of Firmicutes in the oysters from Zhuhai reached the highest bacterial community composition, up to 43.62%, whereas they accounted for only 4.47% in the oysters collected from Guangzhou. However, this trend showed reverse results when the abundance of Bacteroidetes was investigated. Bacteroidetes constituted the largest proportion of the bacterial community, up to 36.98%, in the oysters collected from Guangzhou; however, they accounted for only 9.57% in the oysters collected from Zhuhai. The variable tendencies of Tenericutes obtained from Guangzhou to Jiangmen to Zhuhai were the same as those of Bacteroidetes; however, Tenericutes represented a lower community abundance than Bacteroidetes. At the genus level, the most variable genera were *Lactococcus*, *Vibrio*, and *Aeromonas*. *Lactococcus* and *Vibrio* constituted the largest proportions in Zhuhai (28.90 and 28.44%, respectively), whereas *Aeromonas* accounted for a higher proportion in Jiangmen (15.68%) (Supplementary Figure 2). Notably, an important undetermined genus accounted for 19.72% bacterial abundance in samples collected from Guangzhou, and the presence of another unidentified genus was found to be consistent among the genera isolated from samples collected from three cities (Supplementary Figure 2). The results showed that the dominant microbes were highly variable. Therefore, we examined the detection rates of various genera in the samples. The heatmap indicated that *Vibrio*, *Shewanella*, *Aeromonas*, *Mycoplasma*, and *Acinetobacter* were the most prevalent and chief genera present in the oysters, while *Ralstonia, Ochrobactrum*, *Clostridium*, *Streptococcus*, and *Sphaerochaeta* displayed relatively high abundance levels but were not present in all samples (Figure 3B).

### Functional Properties of the Bacterial Community

**FIGURE 4:**
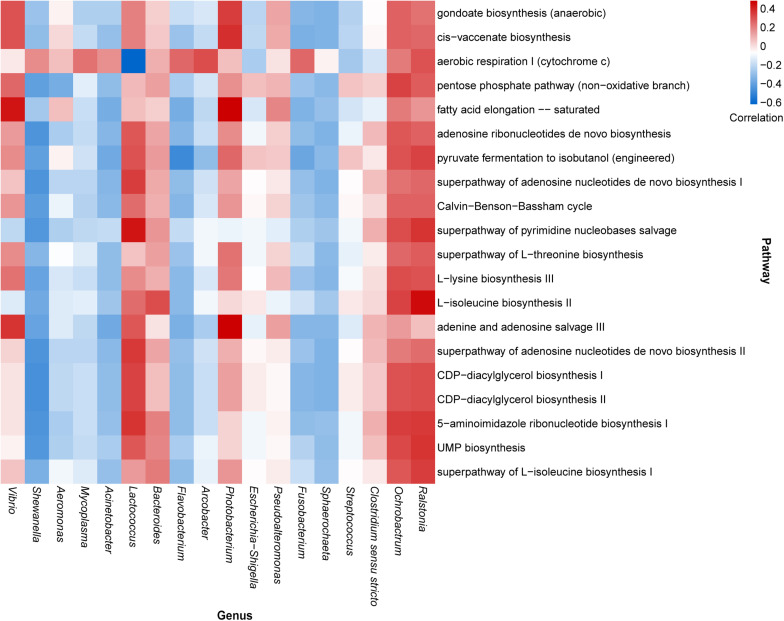
Heatmap illustrating the correlation between the highly abundant genera and top enriched metabolic pathways in oyster samples.

According to the composition of the bacterial community investigated herein, the functional properties of the bacterial community were determined across all samples using PICRUSt2 (Supplementary Table 3). We then analyzed the correlation between highly abundant genera and the top annotated metabolic pathways. The results predicted that the core dominant genus, *Vibrio*, was positively correlated with fatty acid elongation, adenine, and adenosine salvage III pathways; however, *Shewanella*, the second most abundant genus, showed a negative correlation with most of the top annotated metabolic pathways. Similar negative correlation patterns were also observed for the genera *Mycoplasma*, *Acinetobacter*, *Flavobacterium*, *Fusobacterium*, and *Sphaerochaeta*. Additionally, *Lactococcus* showed a strong positive correlation with the pathway of pyrimidine nucleobase salvage and a negative correlation with aerobic respiration I (cytochrome c). Notably, although the genera *Ralstonia* and *Ochrobactrum*had relatively lower detectable rates, they exhibited remarkable correlations with many metabolic pathways (Figure 4).

### Interaction Network of Potentially Pathogenic Bacteria and Other Bacteria in Oysters

**FIGURE 5:**
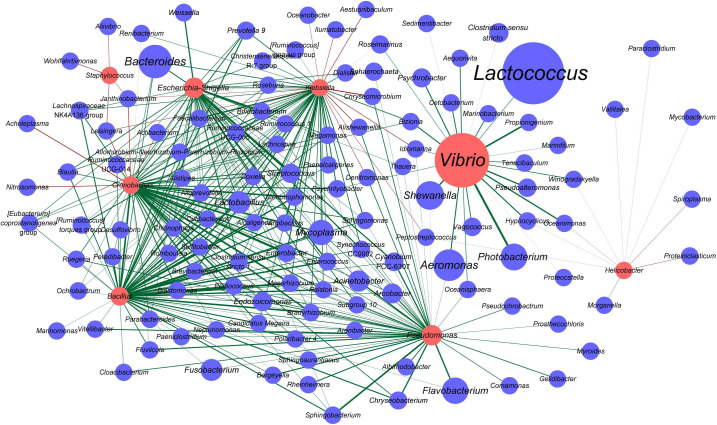
Interaction network of the genera harboring potentially pathogenic species and other bacteria in oysters. The nodes represent taxa at the genus level, and the edges represent correlations between taxa pairs. The nodes in red indicate potentially pathogenic genera. The sizes of the nodes vary according to the total abundance of the corresponding genus in the oyster samples. The edges are colored based on the correlation types, with green indicating positive correlations and red indicating negative correlations.

*Vibrio*, *Staphylococcus*, *Escherichia*-*Shigella*, *Cronobacter*, *Bacillus*, *Klebsiella*, *Pseudomonas*, and *Helicobacter* are the genera that are known to include certain pathogenic species. Also, some OTUs belonged to these genera which were indeed highly homologous to the notorious pathogenic species in corresponding genus based on the phylogenetic analysis (Supplementary Figures 3–10). In the samples analyzed herein, pathogenic *Vibrio* species, such as *V. parahaemolyticus*, *V. fluvialis*, *V. anguillarum*, *V. vulnificus*, *V. cholerae*, *V. fortis*, and *V. metschnikovii*, were detected (Supplementary Figure 3). Among them, the prevalence of *V. anguillarum* was the highest (35/52), followed by *V. parahaemolyticus* (23/52), *V. vulnificus* (13/52), and *V. metschnikovii* (3/52). *V. fluvialis* and *V. cholerae* were detected in one sample (1/52) (Supplementary Table 2). We then constructed an interaction network between the genera potentially harboring pathogenic species and all other genera. Accordingly, *Vibrio* was found to establish marked interactions with *Photobacterium*, followed by *Propionigenium*, and these interactions were positively correlated (Figure 5). Furthermore, *Vibrio* was positively correlated with *Lactococcus*, one of the most abundant genera observed in oyster samples. *Bacteroides*, the genus with higher relative abundance, was positively correlated with *Cronobacter*, *Bacillus*, *Escherichia*-*Shigella*, and *Klebsiella. Cronobacter* and *Klebsiella* were positively correlated with many genera; however, they were also negatively correlated with a few genera, such as *Janthinobacterium* and *Leisingera*. Additionally, *Janthinobacterium* and *Leisingera* were negatively correlated with *Staphylococcus.* Notably, the interactions of *Staphylococcus* and *Helicobacter* with other bacteria in oysters showed negative correlation (Figure 5).

## Discussion

Since oysters are typically reared in uncontrolled, and often dynamic, coastal and estuarine environments, prediction, management, and control of their microbial communities poses challenges (King et al., 2019). Depending on the environment in which oysters are reared, they are subject to different microbial infections, which can also be affected by human activities (Guo and Ford, 2016). The microbial criteria for obtaining satisfactory-quality oysters at wholesale have been set at 5.7–6.2 log CFU/g by Australia and New Zealand and the U.S. Food and Drug Administration (US Food and Drug Administration, 2007; Food Standards Australia New Zealand, 2011; Fernandez-Piquer et al., 2013). In addition, the TVCs in the retail oyster samples collected from the Guangdong area were lower than the reported oysters collected in other places. The TVCs in the Pacific oysters harvested by a commercial grower in Pipeclay Lagoon, Tasmania, Australia, fluctuate between 4 and 6 log CFU/g (Fernandez-Piquer et al., 2011). And in the Pacific oysters harvested in Tasmania, Australia, the mean TVCs predicted in the short supply chain were greater than 6 log CFU/g (Fernandez-Piquer et al., 2013). Therefore, the TVCs in the collected oyster samples in our study were acceptable (Figure 1). All indices presented in Figure 2A jointly indicated that there was a high bacterial diversity of oysters in the Guangdong area. Compared with the oyster samples from JM and ZH, the samples from GZ showed higher microbial richness and diversity. The richness and diversity could be influenced by many factors including growing environment and storage (Trabal et al., 2012; Chen et al., 2017). The raw oysters in Guangzhou seafood market are transported from other places, while the oysters sold in JM and ZH markets are local. The oysters sold in GZ underwent a longer storage, and their growing environment were unknown. These may be the reasons for the higher abundance and diversity of microorganisms in these oysters.

Proteobacteria was the dominant phylum in samples collected from all three cities. Among the five genera with the highest prevalence in oyster samples from the Guangdong region, *Vibrio*, *Shewanella*, *Aeromonas*, and *Acinetobacter* belong to the phylum Proteobacteria. Notably, *Shewanella* is spoilage-causing bacteria present in iced fresh seafood (Gram and Huss, 1996), and the higher abundance of this genera in samples of Guangzhou indicated that the transport process might promoter the growth of spoilage bacteria (Supplementary Figure 2). Additionally, the genera of the bacteria identified in the samples, including *Escherichia*-*Shigella*, *Photobacterium*, *Arcobacter*, *Pseudoalteromonas*, and *Ochrobactrum*, also belong to Proteobacteria. Firmicutes was the dominant phylum in oyster samples from Guangzhou and Jiangmen. *Lactococcus*, *Streptococcus*, and *Clostridium sensustricto* were identified in the samples belonging to Firmicutes. Bacteroidetes was the dominant phylum in oysters collected in Guangzhou and Zhuhai. *Bacteroides* and *Flavobacterium* present in the samples belong to the phylum Bacteroidetes. These three phyla were also the main phyla present in oysters in previous reports (Math et al., 2010; Adams et al., 2019; Chen et al., 2019).

In fact, most of the bacterial isolates detected in our samples were typically obtained from oysters. Some isolates are human pathogens, and some others accelerate oyster spoilage. The most abundant species in our sample belonged to the genus *Vibrio*. Members of the genus *Vibrio*, usually found in oysters, are natural inhabitants of the marine environment (Prapaiwong et al., 2009). Although many *Vibrio* species are harmless to humans, few species can cause diseases in humans (Froelich and Noble, 2016). Especially, the major pathogenic *Vibrio* species, *V. vulnificus*, which can cause sepsis and wound infection, was detected in multiple samples. We also detected several OTUs homologous to *V. parahaemolyticus* according to the phylogenetic analysis (Supplementary Figure 3). The presence of these two *Vibrio* sp. in oysters were occasionally reported (Cook et al., 2002; Kaufman et al., 2003; Jones et al., 2014). Although not all of the *V. parahaemolyticus* and *V. vulnificus* strains are pathogenic to humans, the presence of important virulence genes in the two *Vibrio* sp. isolated from oysters could reach a relatively high frequency in some cases (>50%) (DePaola et al., 2003; Turner et al., 2014; Davis et al., 2021). Climate change and rising sea temperatures that favor the spread of *Vibrio* were also thought to be partly responsible for the rise in vibriosis (Baker-Austin et al., 2018). Particularly, the prevalence of the *thermostable direct hemolysin* (*tdh*) and *thermostable direct-related hemolysin* (*trh*) genes in *V. parahaemolyticus* was found to increase with temperature (Turner et al., 2014). Davis et al. also found that the prominent pathogenic *V. parahaemolyticus* strains in South Puget Sound flourish with exposure to relatively warm temperature (Davis et al., 2021). Our samples were collected in mid-October 2020 (Supplementary Table 1), and the average temperature in Guangdong region during this period was 23.7°C, which was considered to be relatively suitable for the growth of *Vibrios* (Turner et al., 2014). In addition, although short-term storage at low temperatures can limit bacterial growth in oysters (Spaur et al., 2020), *V. parahaemolyticus* will not thoroughly death and can recover their viability in appropriate situation (Xie et al., 2019). This may partially explain the observation that the oysters sold in Guangzhou city still showed high abundance of *V. parahaemolyticus* and *V. vulnificus* after a period of storage (Supplementary Table 2). Thus, further survey targeting the occurrence of known virulence factors for *V. parahaemolyticus* or *V. Vulnificus* and their viable densities is needed to exactly clarify the health risk from these two *Vibrio* sp.

Besides, *V. cholerae*, *V. anguillarum*, *V. fluvialis*, and *V. furnissii*, which can cause diarrhea or other human infections (Chowdhury et al., 2013; Ballal et al., 2017; Baker-Austin et al., 2018; Sinatra and Colby, 2018), were also found in a small number of oyster samples. *V. cholerae* has become the most studied *Vibrio* due to the serious impact of cholera on human health caused by it (Faruque et al., 1998). Patients with diarrhea due to the infection of *V. cholerae* often die if they do not receive prompt treatment (Chen et al., 2020). Although *V. metschnikovii*is were rarely observed in human infections, it has been associated with a few cases of sepsis, wound infection, cholecystitis, and pneumonia (Jean-Jacques et al., 1981; Hansen et al., 1993; Linde et al., 2004; Wallet et al., 2005). A number of virulence factors make *Vibrio* infections fatal to humans. Bacterial hemolysins have been identified as important virulence factors of *Vibrio* due to their contribution to hemorrhagic septicemia (Zhang and Austin, 2005). Cholera toxin is important for the severe diarrhea caused by *V. cholerae* (Rivera-Chávez and Mekalanos, 2019). Therefore, the assessment of the presence of pathogenic *Vibrio* sp. was considered necessary due to the increasing importance of some related infections (Koh et al., 1994; Tantillo et al., 2004).

In addition to *Vibrio*, other microbial genera identified in the samples also include species that are pathogenic to humans and often cause a variety of infections. *Acinetobacter* species are non-fermentative, gram-negative coccobacilli that are ubiquitous in the environment. A few species of this genus reportedly cause human infections, including *A. radioresistens*, *A. calcoaceticus*, and *A. lwoffii* (Rathinavelu et al., 2003; Nonaka et al., 2014; Wang et al., 2019). *Bacteroides fragilis* is one of the most prevalent members of the genus *Bacteroides* has been reported as a common opportunistic pathogen in clinical infections. It may cause a range of diseases involving a permeable intestinal barrier (Sun et al., 2019). *Arcobacter* species are considered emerging gastrointestinal pathogens (Van den Abeele et al., 2018). *A. skirrowii* is a gram-negative pathogenic microorganism that is abundant in various aquatic environments (Morita et al., 2004; Ertas et al., 2010). It is responsible for diseases, including watery diarrhea and septicemia in humans (Oliveira et al., 2018). Regarding other genera, although we did not find a specific disease-causing microbial species, there were a few species in the samples that could not be identified, and these unknown microbes could pose a potential health risk to humans. For example, in these samples, abundance of *Aeromonas* and *Escherichia*-*Shigella* was observed, and species of these genera cannot be easily distinguished from one another. *Aeromonas* species can cause a variety of infections, including sepsis and gastrointestinal diseases (Altwegg and Geiss, 1989). *Escherichia*-*Shigella*is the gram-negative bacterium that can cause bacillary dysentery (shigellosis) in humans (Belotserkovsky and Sansonetti, 2018).

Certain core genera may be correlated with metabolic pathways in oysters. For example, certain *Vibrio* species reportedly exhibit a strong ability to produce long-chain fatty acids (Massengo-Tiassé and Cronan, 2008; Estupiñán et al., 2020), as evidenced by the correlation analysis presented in Figure 4. *Vibrio* has also been correlated with adenine and adenosine salvage pathways and the recycling of adenine and adenosine into adenosine triphosphates (Schuster and Kenanov, 2005). It has been reported that *V. parahaemolyticus* can use adenosine produced by the hydrolysis of nucleotides (Sakai et al., 1987). Although *Shewanella*, *Mycoplasma*, *Acinetobacter*, *Flavobacterium*, and *Fusobacterium* were negatively correlated with most of the metabolic pathways, they were markedly correlated with aerobic respiration I (cytochrome c). Cytochrome c oxidase plays a central role in aerobic respiration, which is an efficient energy-producing metabolic process (Tosha and Shiro, 2013). This reflects the active energy metabolism of these microorganisms. *Lactococcus* is a facultative anaerobic bacterium. Although it showed a negative correlation with aerobic respiration I, other energy metabolism pathways might also provide energy for the process. The most studied species of *Lactococcus* is *L. lactis*, which can be used to perform fermentation in the food industry and can be used in medical engineering (Liu et al., 2019). *L. lactis* was not identified in our samples, but there was a considerable number of unknown *Lactococcus* species in our sample. As *Lactococcus* exhibited a strong correlation with many metabolic pathways, including the biosynthesis of a variety of substances (Figure 4), and as there was a presence of *L. hircilactis* that might be used as aromatic cultures in cheesemaking (Tidona et al., 2018), we suggest that identification of new *Lactococcus* species that synthesize specific metabolites can be achieved using the isolates obtained in the present study and can be used in food fermentation processes.

Interestingly, according to the co-occurrence network analyzed herein (Figure 5), *Vibrio* and *Lactococcus*, the two dominant bacteria in oysters, were found to co-occur. Other microorganisms that establish interactions with *Vibrio* are not mutually exclusive to *Vibrio*. These results showed that the dominant *Vibrio* population might be symbiotic with a wide range of bacterial genera in oysters, and microorganisms that were negatively correlated with *Vibrio* remain to be identified. We suggest that this may also be one of the contributing factors to *Vibrio* exhibiting predominance in oysters.

## Conclusion

In conclusion, a variety of microorganisms were detected in retail Pacific oysters collected in Guangdong, China. The dominant bacteria in oysters belonged to the genus *Vibrio*, which includes a variety of potentially pathogenic bacteria. Thus, the detection of pathogenic bacteria, in particular the pathogenic *Vibrio* sp., as well as their viable densities and the presence of their virulence factors, is necessary before oysters entering the consumer market. In addition, it will be useful to establish a certification program in oyster farming areas that classifies oysters based on microbial testing results. These measures can help to ensure food security when consuming raw oysters. In addition, certain microbes in other genera may also cause intestinal diseases in humans, several measures can be implemented to reduce the risk of infection. Most bacteria, including those belonging to the genus *Vibrio*, cannot survive at high temperatures. Compared to raw seafood, *Vibrio* species in fully cooked seafood were less frequently detected (Monsreal et al., 2015). Thus, oyster consumers are advised to avoid eating raw oysters to reduce the risk of infection. Oyster processors should also avoid wound contact with oysters.

## Data Availability Statement

The data presented in the study are deposited in the NCBI repository, accession number PRJNA736520.

## Author Contributions

MY designed and supervised the entire study, helped in microbial diversity analysis and microbiome interaction analysis, and wrote the manuscript. XW carried out sample collection and DNA extraction. AY carried out microbiological assays. All authors read and commented on the manuscript.

## Conflict of Interest

The authors declare that the research was conducted in the absence of any commercial or financial relationships that could be construed as a potential conflict of interest.

## Supplementary Material

Supplementary Figure 1 The sampling sites of oyster samples in this study.

Supplementary Figure 2 The relative abundance species of bacteria at the genus level.

Supplementary Figure 3 Maximum likelihood phylogeny of OTUs belonged to genus *Vibrio* and the notorious pathogenic *Vibrio* species. The pathogenic species are indicated with orange.

Supplementary Figure 4 Maximum likelihood phylogeny of OTUs belonged to genus *Staphylococcus* and the notorious pathogenic *Staphylococcus* species. The pathogenic species are indicated with orange.

Supplementary Figure 5 Maximum likelihood phylogeny of OTUs belonged to genus *Shigella* and the notorious pathogenic *Shigella* species. The pathogenic species are indicated with orange.

Supplementary Figure 6 Maximum likelihood phylogeny of OTUs belonged to genus *Cronobacter* and the notorious pathogenic *Cronobacter* species. The pathogenic species are indicated with orange.

Supplementary Figure 7 Maximum likelihood phylogeny of OTUs belonged to genus *Bacillus* and the notorious pathogenic *Bacillus* species. The pathogenic species are indicated with orange.

Supplementary Figure 8 Maximum likelihood phylogeny of OTUs belonged to genus *Klebsiella* and the notorious pathogenic *Klebsiella* species. The pathogenic species are indicated with orange.

Supplementary Figure 9 Maximum likelihood phylogeny of OTUs belonged to genus *Pseudomonas* and the notorious pathogenic *Pseudomonas* species. The pathogenic species are indicated with orange.

Supplementary Figure 10 Maximum likelihood phylogeny of OTUs belonged to genus *Helicobacter* and the notorious pathogenic *Helicobacter* species. The pathogenic species are indicated with orange.

Supplementary Table 1 The information of sample collection in this study.

Supplementary Table 2 Relative abundance of the observed OTUs among all samples.

Supplementary Table 3 Functional annotations based on the KEGG pathway using PICRUSt 2.
