# Supplementary figures and images for: Microbial Profiles of Retail Pacific Oysters (Crassostrea gigas) From Guangdong Province, China
Source: Front Microbiol. 2021 Jul 7;12:689520. doi: 10.3389/fmicb.2021.689520 (PMC8292972; doi:10.3389/fmicb.2021.689520)

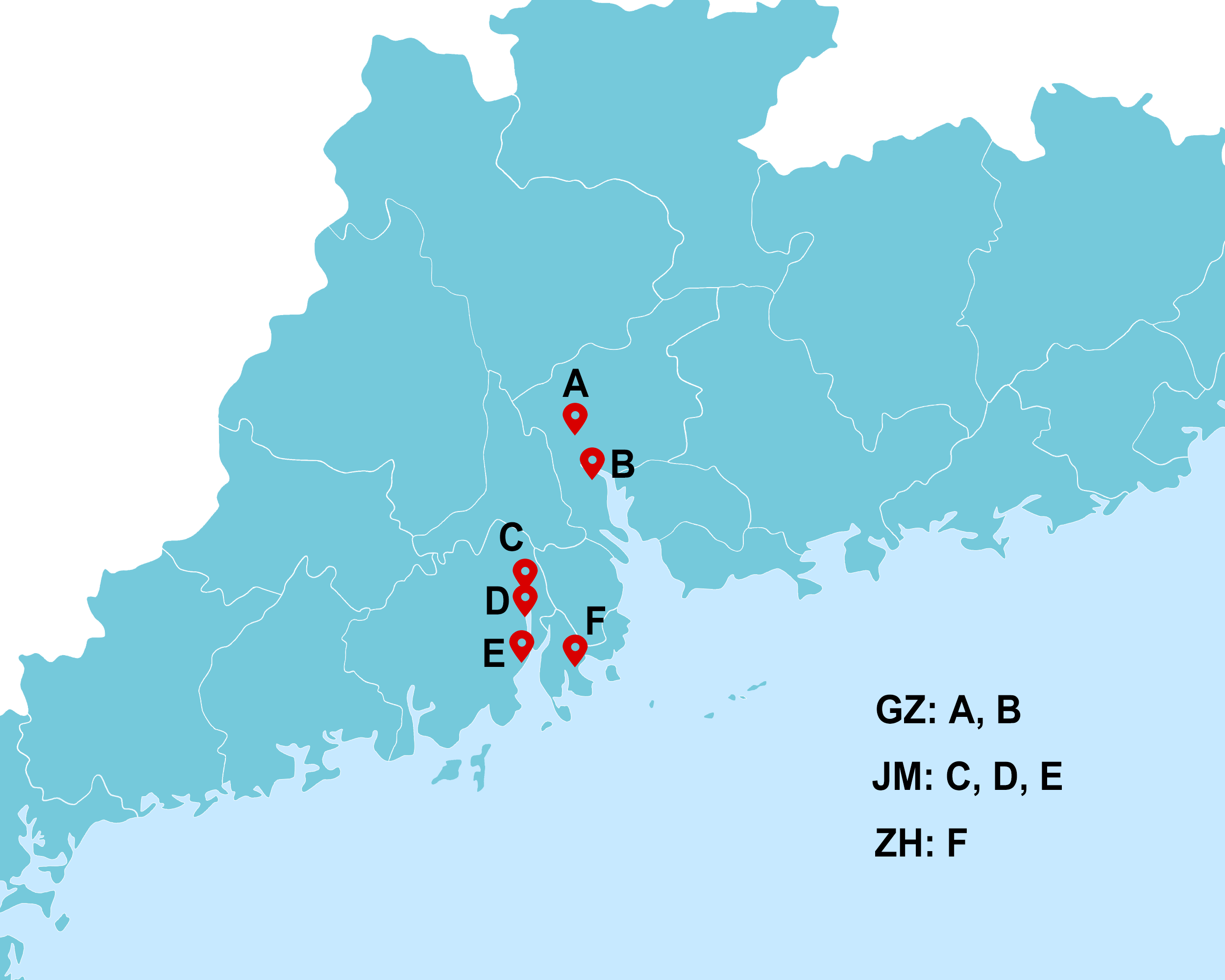

Supplement: Supplementary Figure 1 — The sampling sites of oyster samples in this study. [file Image_1.TIF]

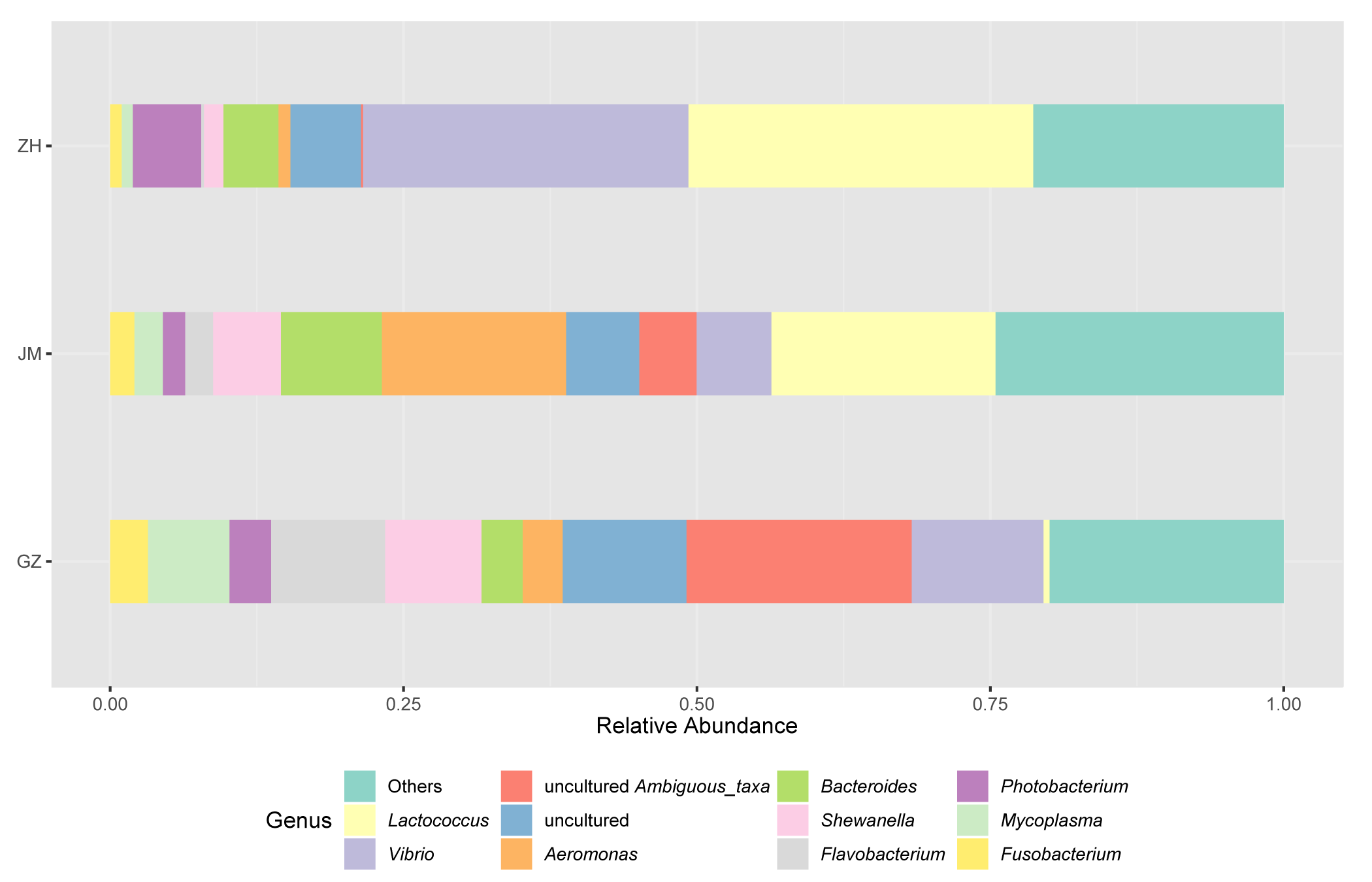

Supplement: Supplementary Figure 2 — The relative abundance species of bacteria at the genus level. [file Image_2.TIF]

Tree scale: 0.1

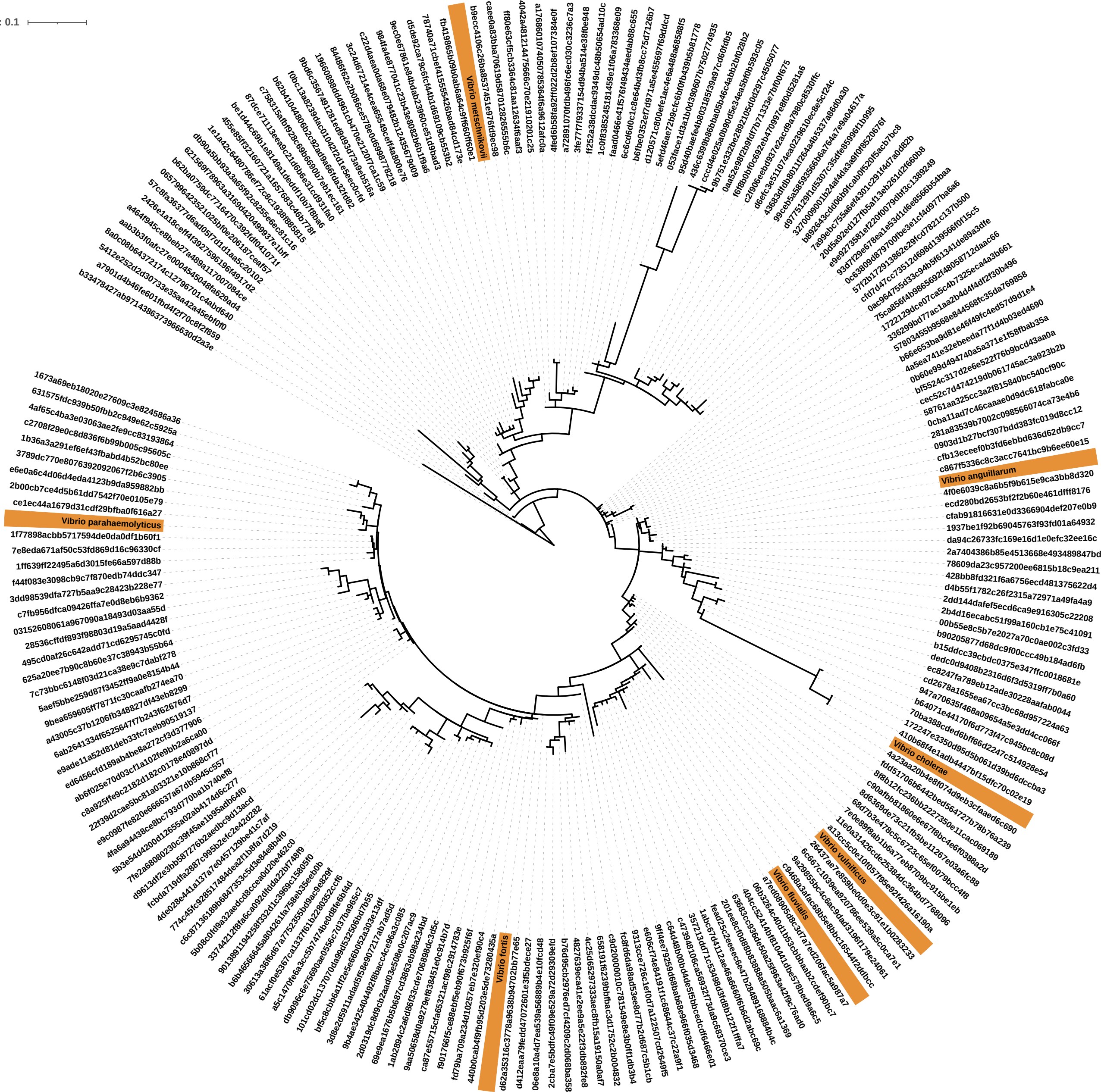

Supplement: Supplementary Figure 3 — Maximum likelihood phylogeny of OTUs belonged to genus Vibrio and the notorious pathogenic Vibrio species. The pathogenic species are indicated with orange. [file Data_Sheet_1.PDF]

Tree scale: 0.01

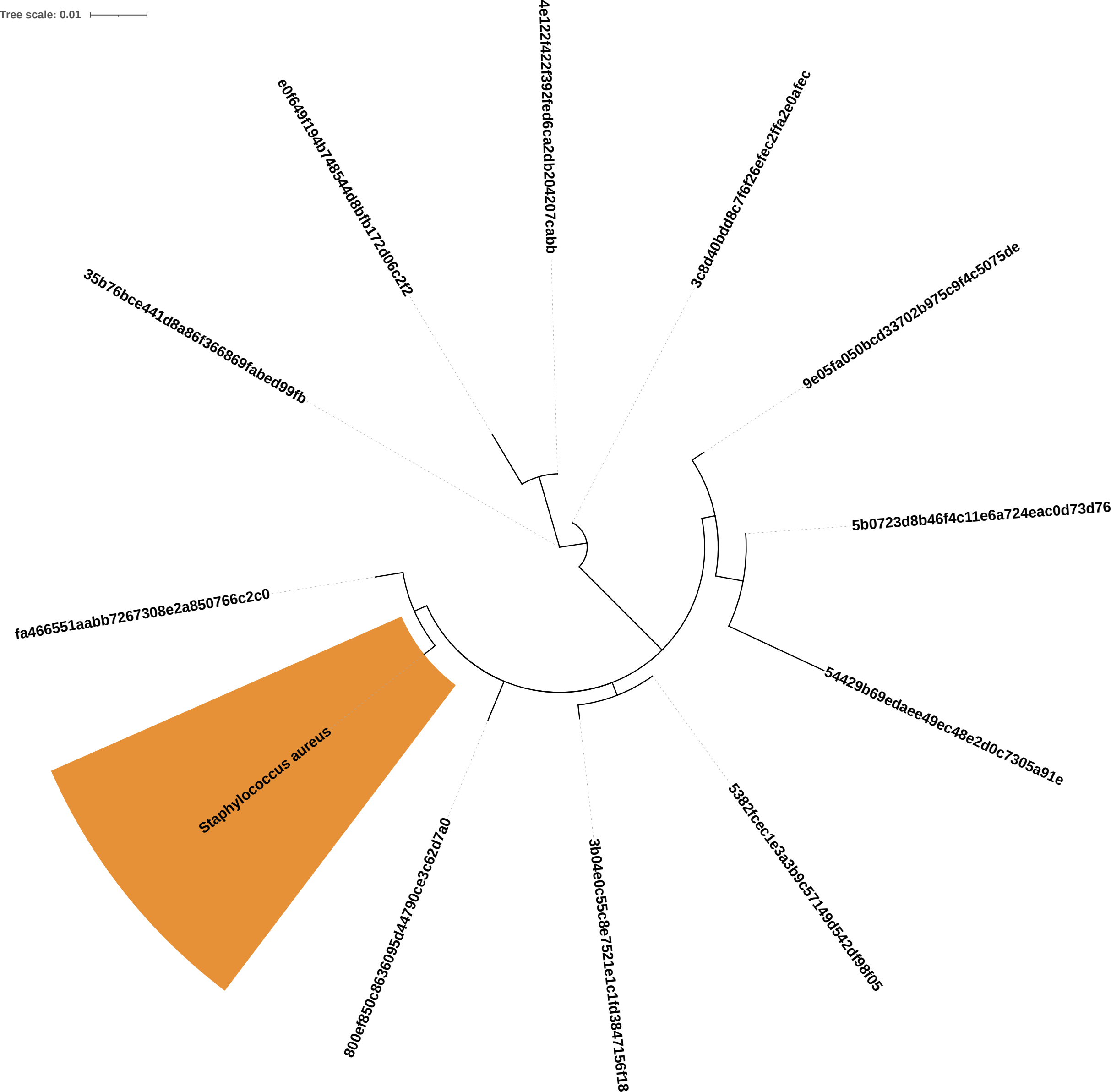

Supplement: Supplementary Figure 4 — Maximum likelihood phylogeny of OTUs belonged to genus Staphylococcus and the notorious pathogenic Staphylococcus species. The pathogenic species are indicated with orange. [file Data_Sheet_2.PDF]

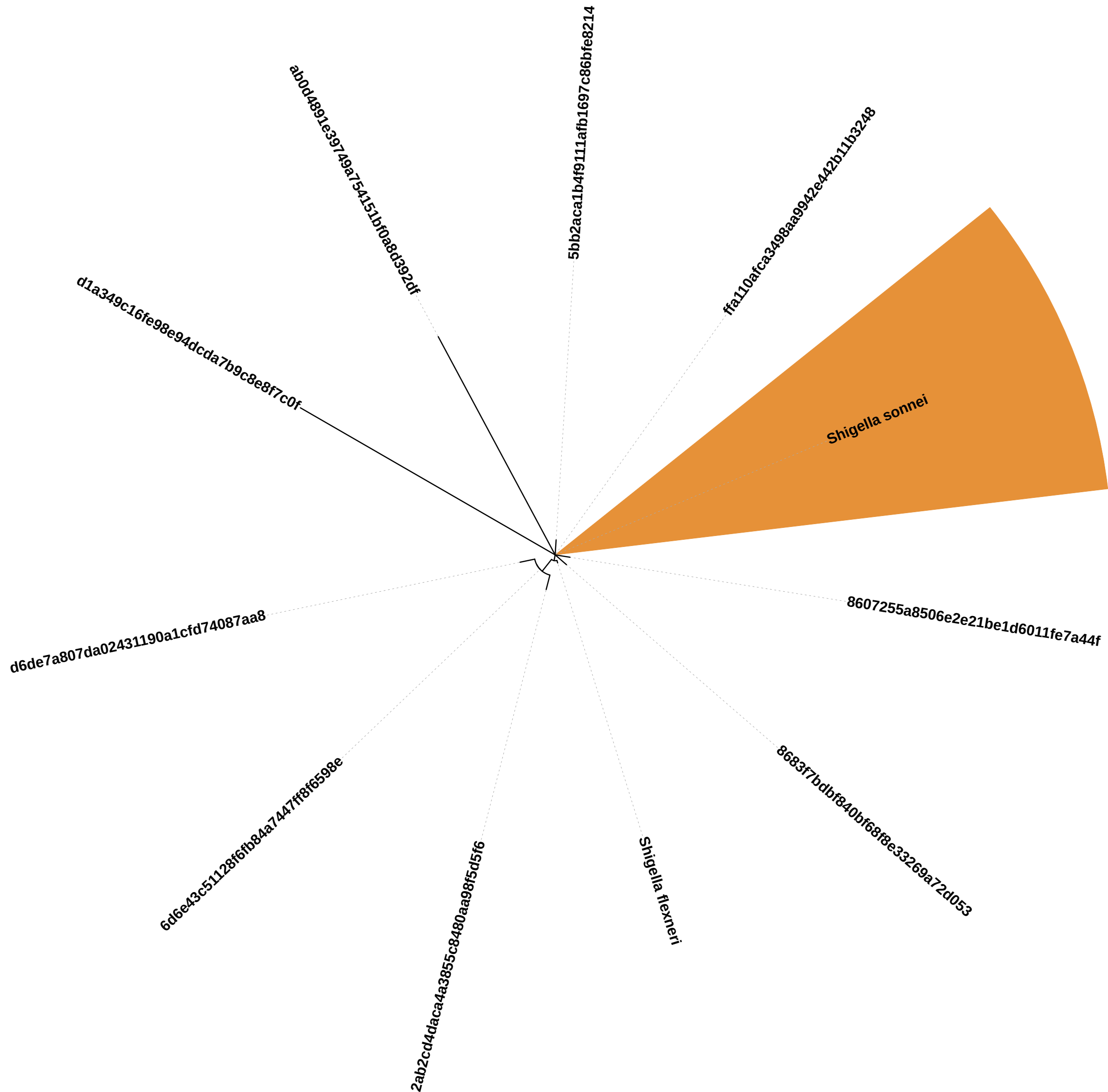

Supplement: Supplementary Figure 5 — Maximum likelihood phylogeny of OTUs belonged to genus Shigella and the notorious pathogenic Shigella species. The pathogenic species are indicated with orange. [file Data_Sheet_3.PDF]

Tree scale: 0.1

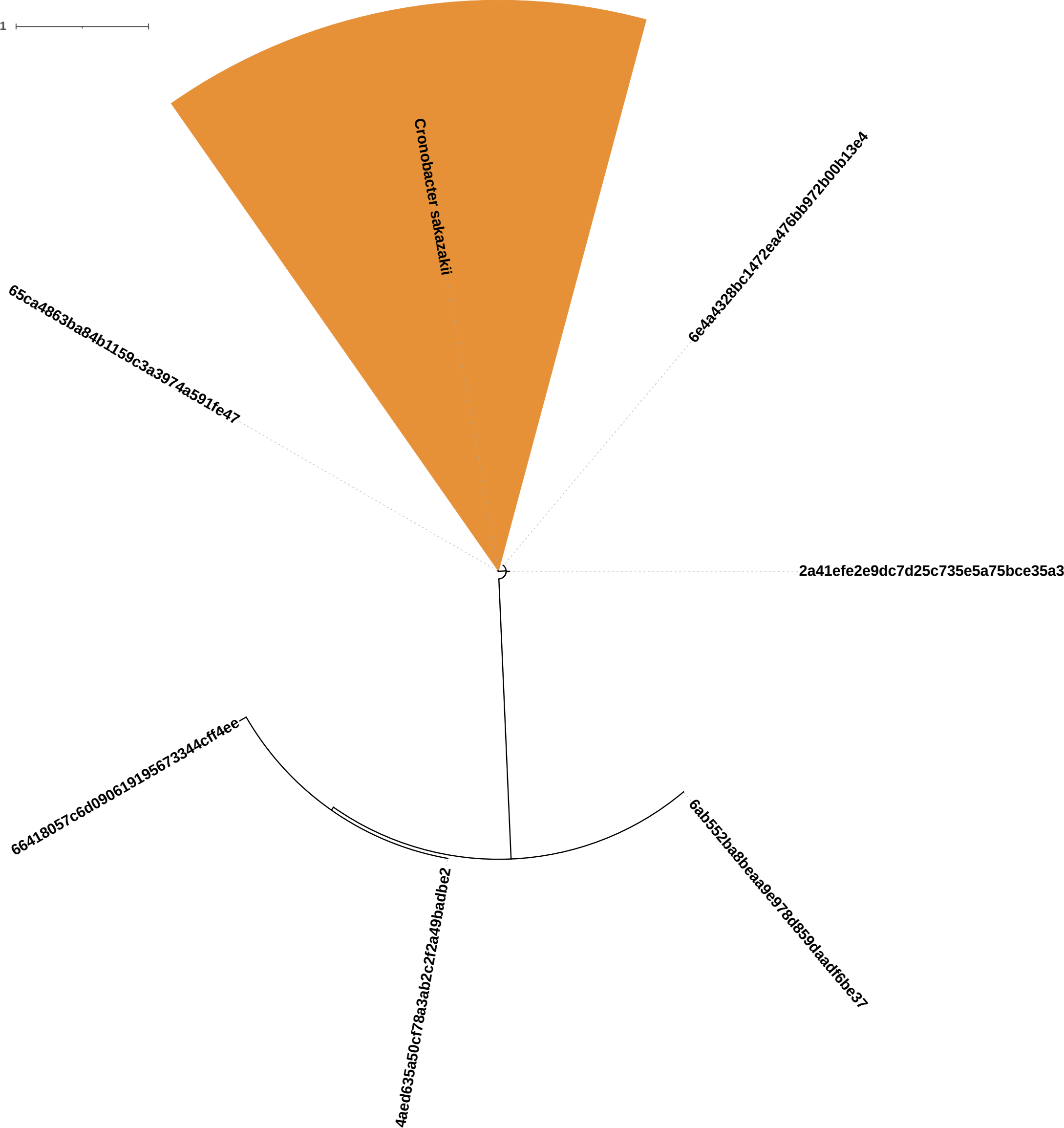

Supplement: Supplementary Figure 6 — Maximum likelihood phylogeny of OTUs belonged to genus Cronobacter and the notorious pathogenic Cronobacter species. The pathogenic species are indicated with orange. [file Data_Sheet_4.PDF]

Tree scale: 0.1

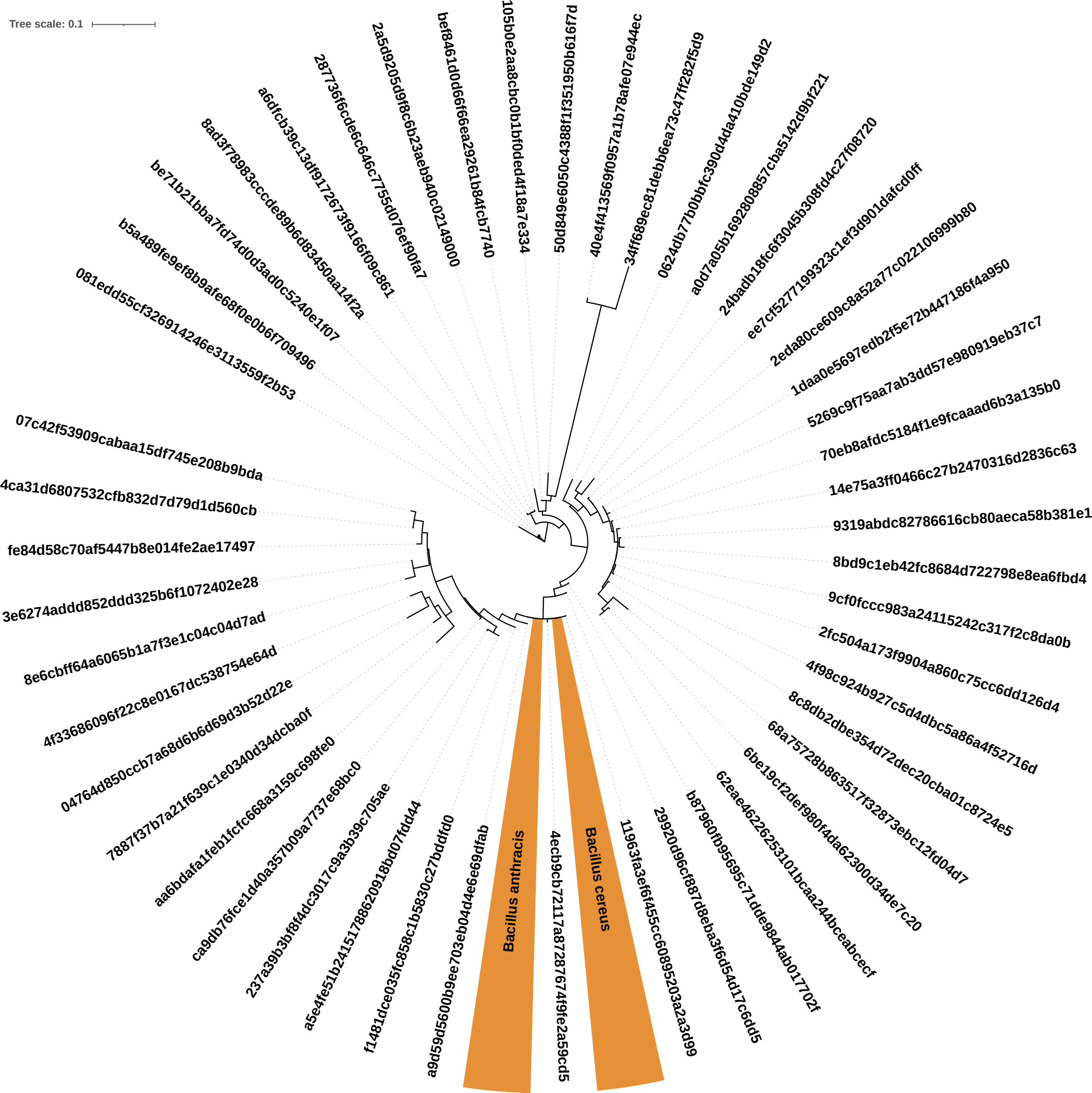

Supplement: Supplementary Figure 7 — Maximum likelihood phylogeny of OTUs belonged to genus Bacillus and the notorious pathogenic Bacillus species. The pathogenic species are indicated with orange. [file Data_Sheet_5.PDF]

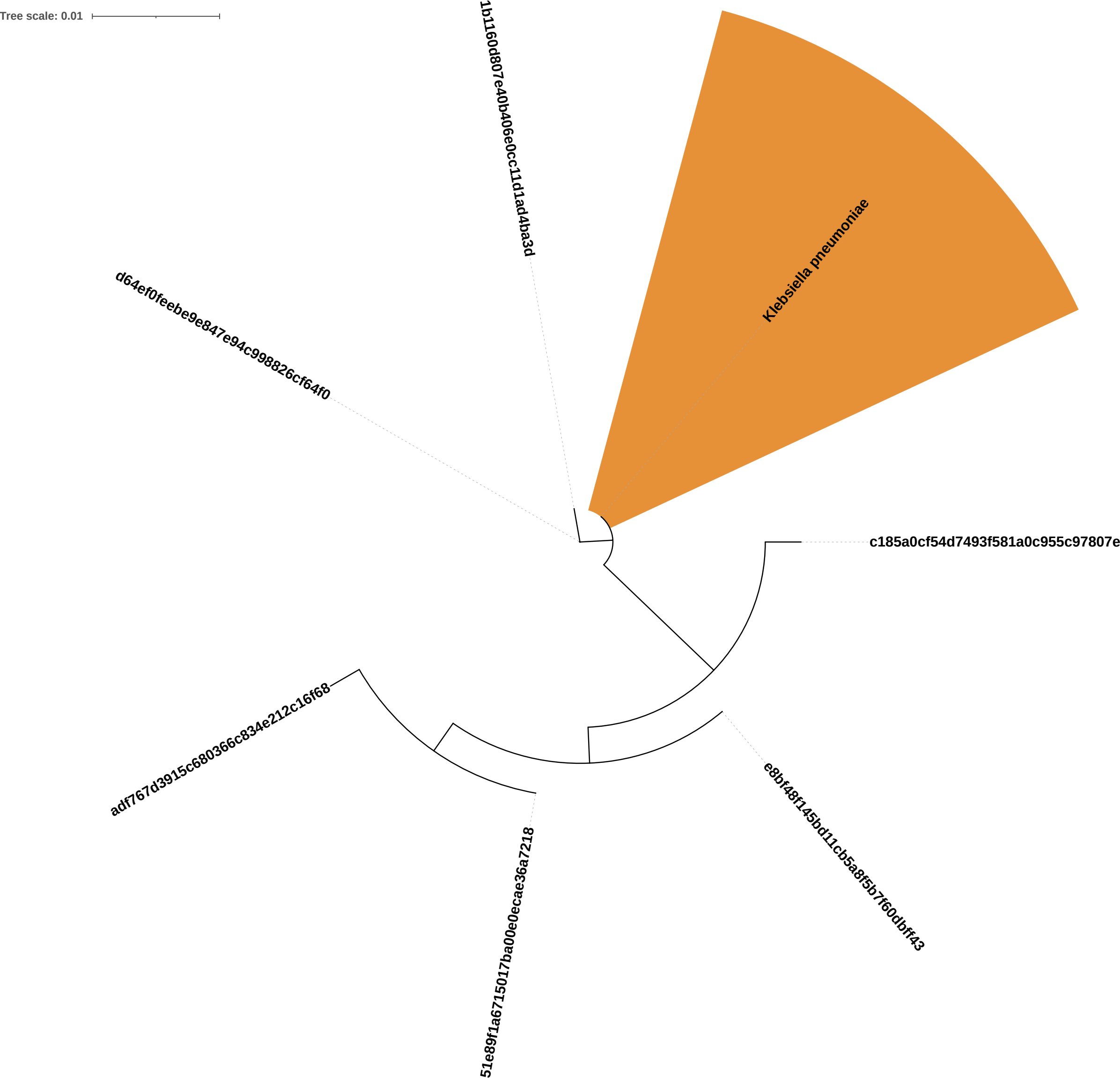

Supplement: Supplementary Figure 8 — Maximum likelihood phylogeny of OTUs belonged to genus Klebsiella and the notorious pathogenic Klebsiella species. The pathogenic species are indicated with orange. [file Data_Sheet_6.PDF]

Tree scale: 0.1

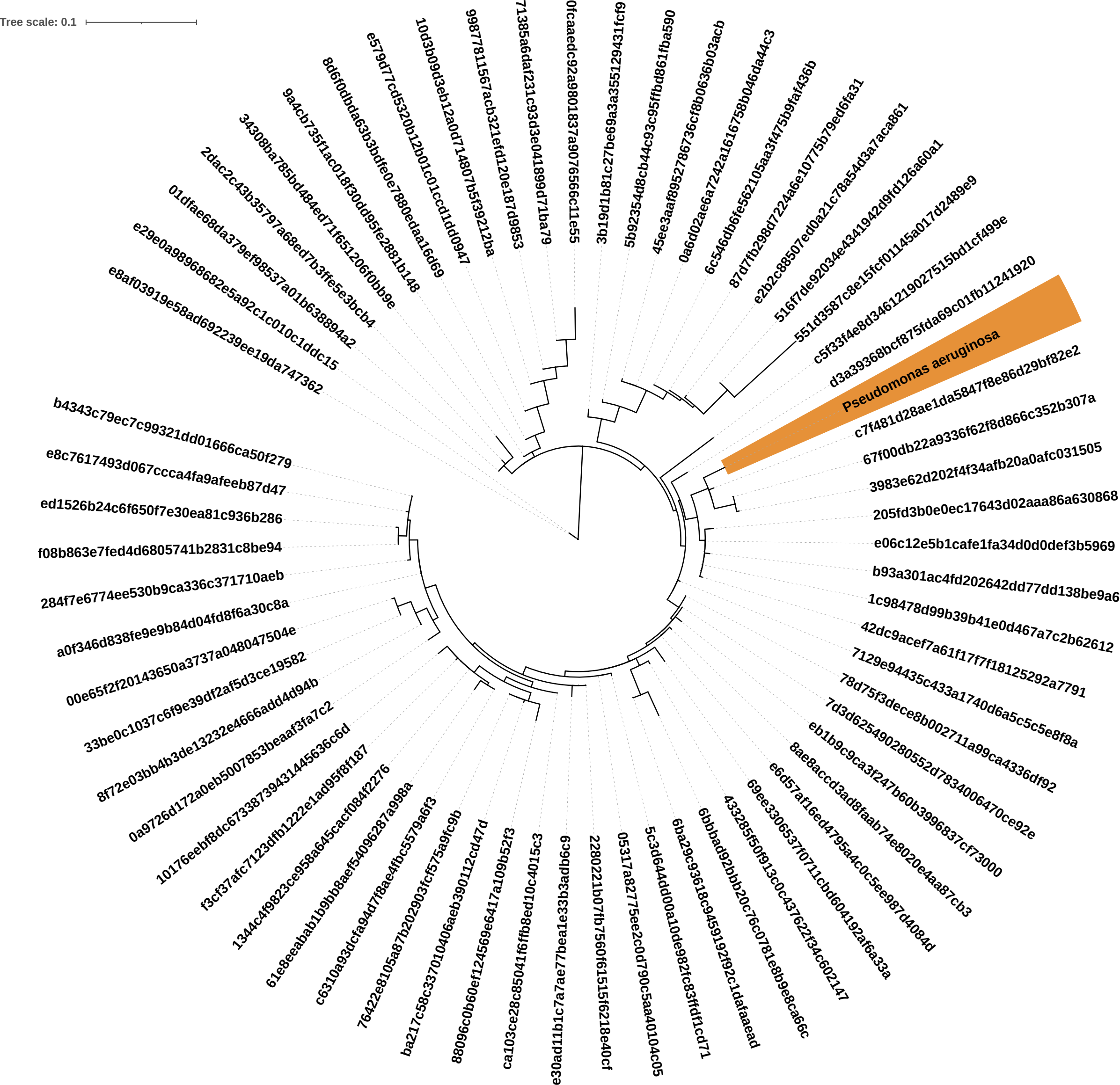

Supplement: Supplementary Figure 9 — Maximum likelihood phylogeny of OTUs belonged to genus Pseudomonas and the notorious pathogenic Pseudomonas species. The pathogenic species are indicated with orange. [file Data_Sheet_7.PDF]

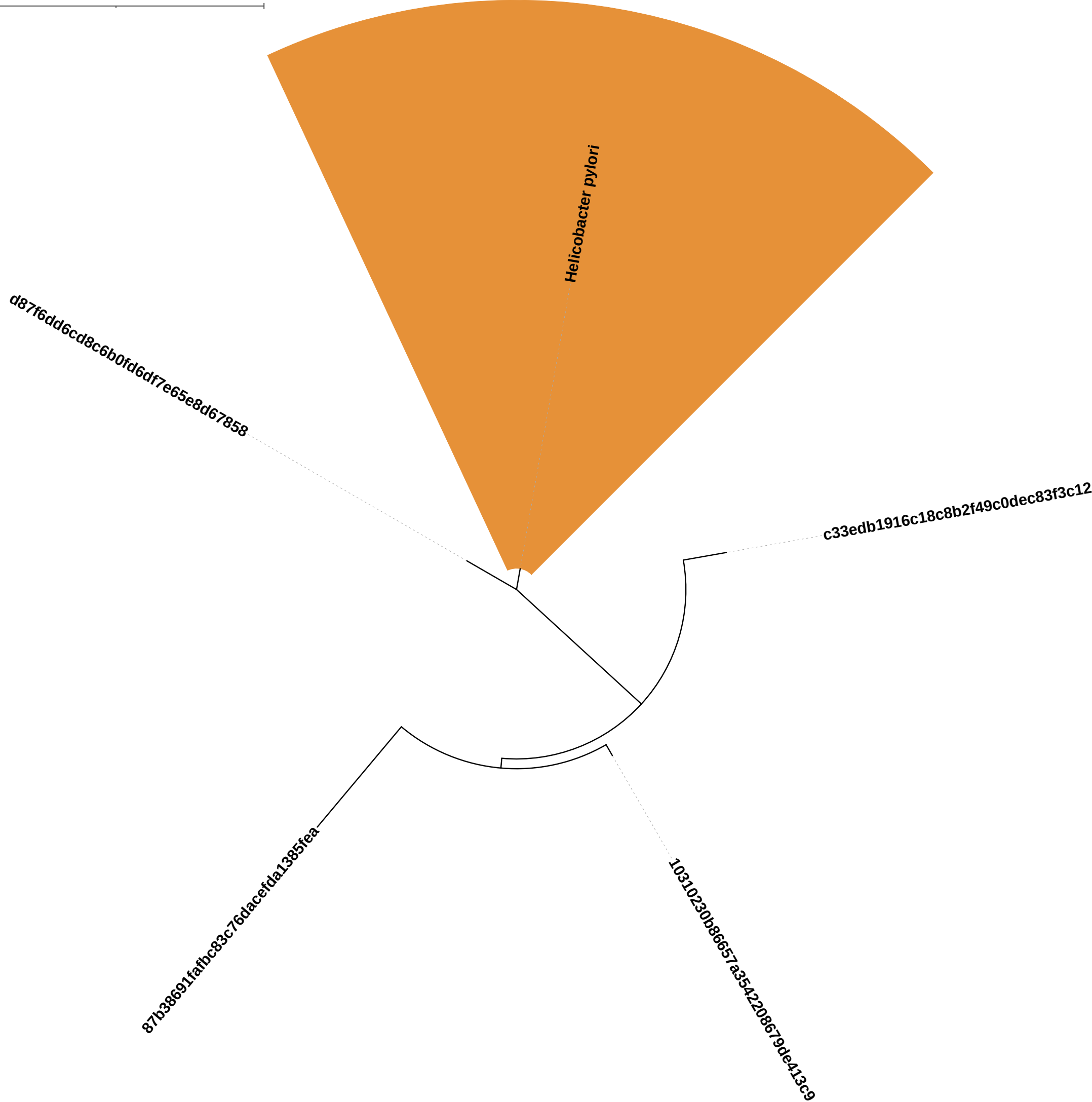

Supplement: Supplementary Figure 10 — Maximum likelihood phylogeny of OTUs belonged to genus Helicobacter and the notorious pathogenic Helicobacter species. The pathogenic species are indicated with orange. [file Data_Sheet_8.PDF]
